# Notch Bandpass Filter with an Independently Controllable Notch Frequency Based on SSPPs and an Annular Slot DGS

**DOI:** 10.3390/mi17030340

**Published:** 2026-03-11

**Authors:** Jinxiao Yang, Shuang Li, Zhongming Kang, Qihao Zhang, Zhe Chen

**Affiliations:** School of Information Science and Engineering, Yunnan University, Kunming 650500, China

**Keywords:** spoof surface plasmon polaritons, notch filter, bandpass filter

## Abstract

In this paper, a notch bandpass filter based on spoof surface plasmon polaritons (SSPPs) is presented and systematically analyzed. The bandpass response is realized by a momentum-matched SSPP transition section and two SSPP resonant units. An annular slot defected ground structure (DGS), evolved from the conventional dumbbell DGS is etched on the ground plane to introduce an in-band notch. The notch frequency can be controlled independently by the DGS geometric parameters while the passband edges remain nearly unchanged. A prototype is fabricated and measured. The measured results agree well with the simulations. Two passbands are obtained from 0.67 to 3.40 GHz and from 3.67 to 4.77 GHz. The insertion loss is 0.48 dB at 2.00 GHz and 1.11 dB at 4.22 GHz. The return loss on both sides of the notch is better than −10 dB. A notch centered at 3.50 GHz provides −25 dB rejection. The compact structure and the independently controllable notch frequency make the proposed filter suitable for narrowband interference suppression in microwave and millimeter-wave front ends.

## 1. Introduction

Surface plasmon polaritons (SPPs) are surface electromagnetic waves supported at a metal–dielectric interface. They propagate along the interface and decay rapidly in the vertical direction normal to the interface. Owing to this field localization, SPPs have been widely exploited in optical devices and systems. At microwave and terahertz frequencies metals are close to perfect conductors, and their permittivity is no longer negative. As a result, natural SPPs cannot be sustained in these lower frequency bands. In 2004 Pendry et al. introduced a periodically structured metallic surface whose dispersion resembles that of optical SPPs [1]. This concept established spoof SPPs, referred to as SSPPs, and stimulated extensive research on low-frequency plasmonic transmission lines and devicess Integrating SSPP structures with microwave passive circuits has since become an active direction. Many components have been reported including bandpass filters [2,3,4,5,6], power dividers [7,8,9,10] and attenuators [11,12].

Since the Federal Communications Commission (FCC) authorized ultra-wideband operation in the 3.1–10.6 GHz range for commercial applications, UWB technology has attracted substantial research interest while introducing new design challenges [13,14]. This spectrum accommodates multiple incumbent wireless services and it is therefore one of the most heavily utilized broadband windows. Typical examples include the 3.5 GHz band for WiMAX, the 5 GHz band for WLAN, the 5.9 GHz band for dedicated short-range communications, and the 8 GHz band used in military and satellite links. Because these services coexist within the UWB allocation, spectral overlap is unavoidable and mutual interference can occur. To ensure link stability and reliability UWB bandpass filters often incorporate notches at selected frequencies to suppress interfering bands or carriers and to mitigate the impact of out-of-band signals on system performance.

Therefore notch filters based on SSPP structures have attracted increasing attention. Owing to the inherent low-pass dispersion of SSPP transmission lines, early efforts mainly introduced notch responses on top of SSPP low-pass prototypes. Notches were realized by etching one or multiple annular slots along the SSPP periodic cells [15,16,17]. Alternative approaches employed annular resonators [18] or embedded H-shaped resonators within the SSPP structure [19]. However, these notch designs are derived from low-pass configurations and noticeable passband ripples are often observed. In practical wireless front ends bandpass filters are more widely used. It is therefore more meaningful to integrate notch functions into SSPP bandpass filters. Xiao et al. achieved a bandpass response by introducing low-frequency suppression using a coplanar waveguide scheme. A notch was then generated by an added resonator to combine notch and bandpass responses [20]. Nevertheless the reported design exhibits relatively high in-band insertion loss and a large footprint which limits miniaturization. Moreover the notch frequency in most existing SSPP-based notch filters cannot be conveniently tuned through SSPP unit cell parameters. This lack of independent controllability reduces design flexibility.

In this paper, a compact notch bandpass filter based on spoof surface plasmon polaritons (SSPPs) is presented and analyzed. A deep notch of 25 dB is achieved at 3.50 GHz to mitigate interference around the 3.5 GHz band which is relevant to several wireless and radar related scenarios. The notch frequency is independently controlled by precisely tuning the key geometrical parameters of the annular slot defected ground structure. A prototype is fabricated and measured to validate the design. Measured results agree well with simulations and confirm the proposed operating mechanism. The proposed filter features low insertion loss, strong notch rejection, compact size and independent notch frequency control during the design stage. It is a promising candidate for narrowband interference suppression in broadband microwave and millimeter-wave front ends.

## 2. Design of SSPP Notch Bandpass Filter

### 2.1. Low-Pass SSPP Unit Design

**Figure 1:**
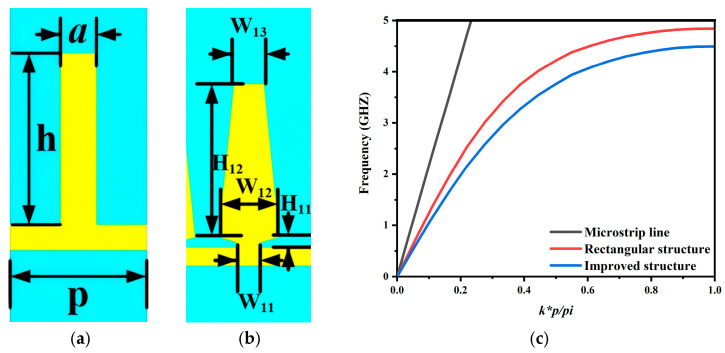
(**a**) Rectangular unit cell, (**b**) improved unit cell, (**c**) dispersion curves.

Figure 1a shows a conventional rectangular SSPP unit cell. Its dispersion follows the periodic metal gap structure theory reported by Harrington [21], as expressed in Equation (1) [22]. In this topology the groove depth h mainly determines the cutoff frequency. Only h and the groove width a provide effective tuning. This limits design flexibility and restricts miniaturization. To address these issues an improved SSPP unit cell is introduced in Figure 1b. It incorporates several additional adjustable geometrical parameters. The dispersion of the proposed unit cell is extracted in CST Microwave Studio using the eigenmode procedure in [23]. The dispersion curve of the microstrip line is plotted as a reference. As shown in Figure 1c, as the frequency increases, the dispersion curves of both unit cell structures gradually deviate from that of the microstrip line and approach their respective asymptotic frequencies at 4.5 GHz and 5 GHz. This behavior clearly indicates the enhancement of field confinement. For the same unit cell height, the improved structure exhibits a lower asymptotic frequency. This feature implies stronger electromagnetic field confinement and demonstrates a greater capability for miniaturization. The dispersion curves in Figure 1c are obtained in CST Microwave Studio. The dispersion characteristics of the SSPP unit cell are evaluated by embedding the proposed cell in an air box. Periodic boundary conditions are applied along the y-direction, while PEC boundaries are imposed on the x- and z-direction faces. Eigen frequencies are then computed by sweeping the phase shift between the two periodic boundaries from 0° to 180°. In this way, the dispersion relation of the fundamental mode is extracted, where k represents the propagation constant along the y-axis.

(1) $$k=k_{0}\sqrt{1+\frac{a^{2}}{p^{2}}tan^{2}(k_{0}h)}$$

**Figure 2:**
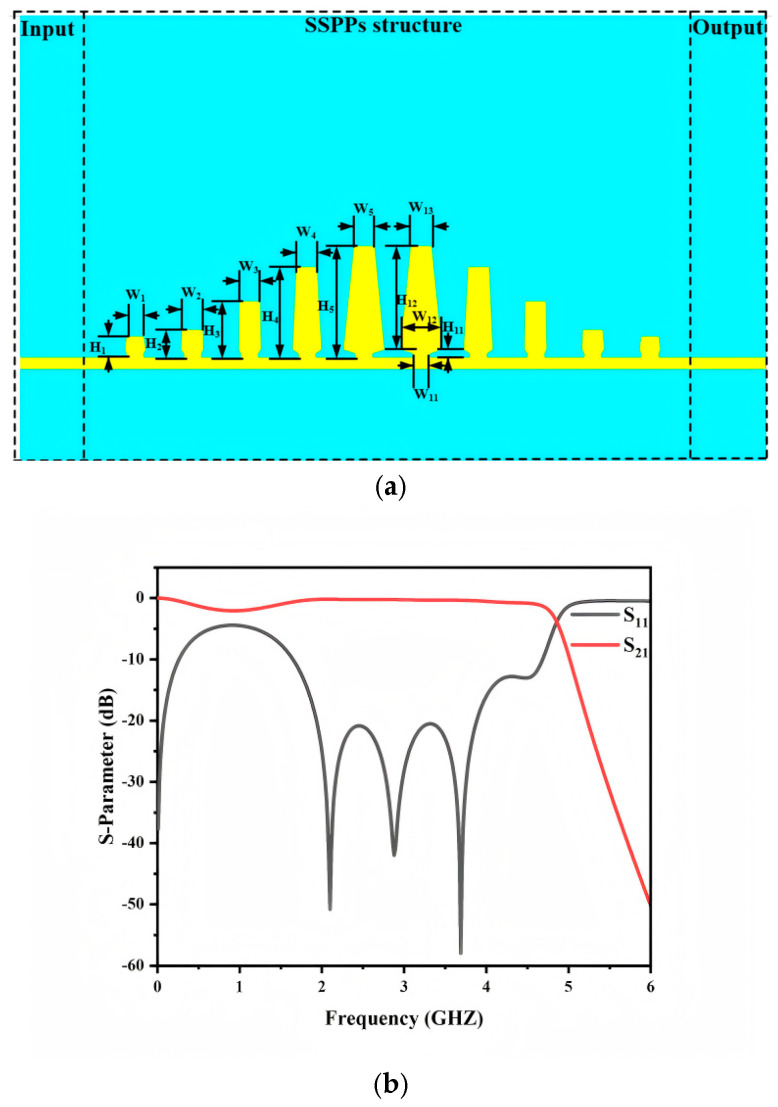
Low−-ss filter based on improved SSPP (**a**) structural layout, (**b**) s-parameters.

Compared with conventional microstrip lines, SSPP transmission lines inherently exhibit a low-pass dispersion. This property provides a natural upper cutoff frequency, which is advantageous for bandpass filter design. By cascading the proposed unit cells with different heights, a low-pass filter is implemented, as shown in Figure 2a. The simulated S-parameters are presented in Figure 2b. The return loss within the passband is generally better than −10 dB, confirming that the improved SSPP unit cell supports a high performance low-pass response. A degraded return loss is observed around 1 GHz. This behavior is mainly attributed to momentum mismatch between the input microstrip port and the SSPP section. In this work, the low-frequency mismatch is acceptable because the subsequent bandpass design intentionally introduces low-frequency suppression. Therefore, the degraded matching around 1 GHz has a limited impact on the overall filter performance.

### 2.2. Design of a Bandpass Filter Based on SSPP Structure

Bandpass filter design requires the establishment of both upper and lower cutoff frequencies. As discussed in Section 2.1, cascading the improved SSPP unit cells with different heights and widths produces a low-pass response. Its cutoff frequency naturally defines the upper band edge and provides effective suppression for undesired signals above the passband. To realize the lower cutoff frequency, a dedicated attenuation mechanism must be introduced at low frequencies. Commonly used methods include using SIW, microstrip-slot-line structures, or short-circuited stubs. The first two approaches increase structural complexity and fabrication difficulty, which can degrade the measured performance of the fabricated prototype. Therefore short-circuited stubs are incorporated into the SSPP unit cells to form resonant units. The resulting resonance introduces low-frequency suppression and establishes the lower cutoff required for the proposed bandpass filter.

**Figure 3:**
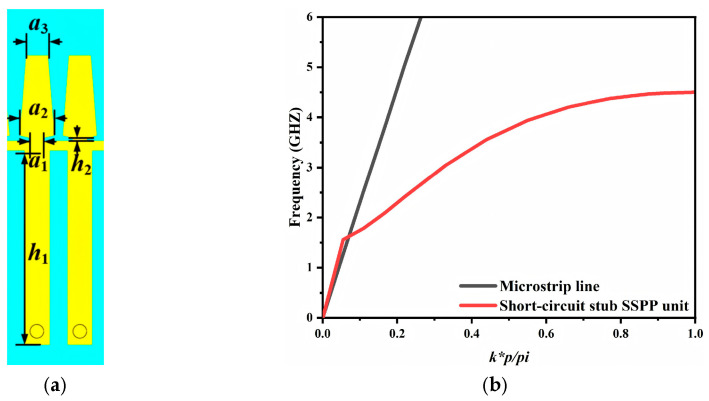
Proposed short-circuit stub SSPP resonator (**a**) structure and (**b**) dispersion curve are shown.

The designed SSPP resonant unit is depicted in Figure 3a. The proposed SSPP cell and the short-circuited stub are symmetrically loaded on the two sides of the main transmission line. The stub is connected to the bottom ground plane through a grounding via, thereby forming the required current return path. The number of SSPP resonant units is also a critical design parameter. Increasing the number of units generally flattens the passband response and improves out of band rejection. However, it also enlarges the circuit footprint and limits the degree of miniaturization. Considering these tradeoffs, two SSPP resonant units are employed in this work. This choice satisfies the required in-band transmission while maintaining a compact overall size.

The dispersion characteristics of a single unit cell are shown in Figure 3b. The dispersion curve of a microstrip line is also plotted for comparison. At low frequencies, the dispersion of the short-circuited SSPP unit cell closely follows the light line and gradually departs from it as frequency increases. It eventually approaches a constant value, referred to as the asymptotic frequency. This asymptotic frequency determines the upper cutoff of the resulting bandpass filter. In addition, an intersection between the two dispersion curves appears in the low-frequency region. This intersection is associated with the lower cutoff frequency. As indicated in Figure 3b, the asymptotic frequency is approximately 4.6 GHz and the intersection frequency is approximately 1.6 GHz. Therefore, based on this short-circuited SSPP unit cell the upper cutoff frequency is expected to be around 4.6 GHz, whereas the lower cutoff frequency is around 1.6 GHz.

**Figure 4:**
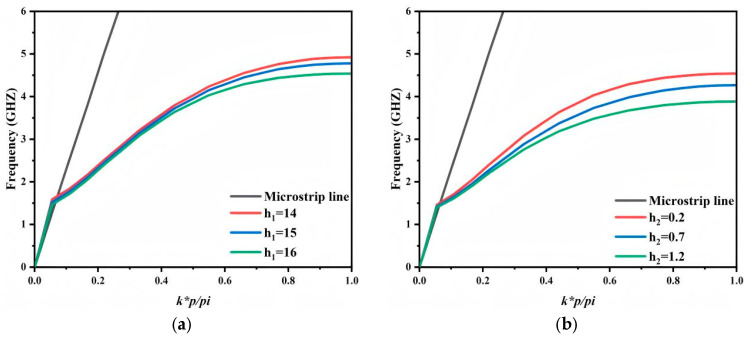
Influence of SSPP unit structural parameters on dispersion curves (**a**) h_1_; (**b**) h_2_.

Furthermore, the proposed short-circuited stub unit cell enables dispersion tailoring through geometric tuning. As shown in Figure 4a,b, varying h_1_ and h_2_ shifts the asymptotic frequency, whereas the intersection point in the low-frequency region remains nearly unchanged. Consequently, the upper cutoff frequency can be flexibly adjusted while the lower cutoff frequency is essentially preserved. As h_1_ and h_2_ increase, the asymptotic frequency decreases and the upper cutoff frequency reduces accordingly. This behavior provides a direct and convenient approach for passband bandwidth adjustment during the design stage.

Based on the above design, the upper and lower cutoff frequencies can be established, thereby yielding a bandpass response. However, the dispersion relation indicates a pronounced momentum mismatch between the microstrip line and the short-circuited stub SSPP unit. If the SSPP resonator is directly connected to the microstrip line, strong passband ripples will occur and additional insertion loss may be introduced, thereby degrading the filter performance. Therefore, a dedicated transition section is required between the microstrip feed and the SSPP resonator to enable smooth mode conversion from the microstrip mode to the SSPP mode while preserving the bandpass characteristics. The low-pass filter introduced in Section 2.1 can be employed as an effective transition unit. It provides momentum matching and a gradual impedance transformation.

**Figure 5:**
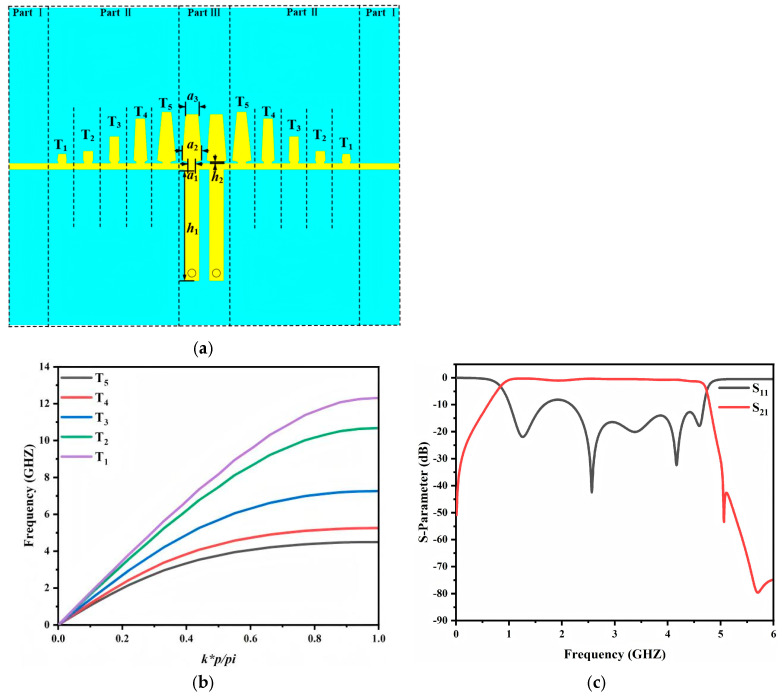
(**a**) Overall circuit structure of the bandpass filter; (**b**) dispersion curve of the transition structure; (**c**) S−parameters of the bandpass filter.

Based on the proposed configuration, the overall bandpass filter layout is illustrated in Figure 5a. The circuit consists of three regions. Region I is the feed section, implemented by a 50 Ω microstrip line with a width of 0.72 mm. Region II is the transition section, which comprises five cascaded units with gradually varied heights and widths. This section is derived from the low-pass SSPP structure in Section 2.1 and provides momentum matching and impedance transformation between the microstrip line and the SSPP resonant transmission line. Region III is the main transmission section, formed by two proposed SSPP resonant units.

Furthermore, the dispersion curves of the transition section are shown in Figure 5b. The asymptotic frequency evolves monotonically from T_1_ to T_5_. It shifts from a value close to that of the microstrip line to a value approaching that of the SSPP resonant unit. This continuous evolution indicates that the proposed transition section provides effective momentum matching between the microstrip mode and the SSPP mode, enabling smooth energy transfer across the transition region and preserving stable in-band transmission. The simulated S-parameters of the overall bandpass filter are plotted in Figure 5c. A clear passband is obtained from 1.0 to 4.6 GHz. The insertion loss remains better than −3 dB over the passband. The filter also exhibits satisfactory out-of-band rejection. This response provides a suitable baseline for the subsequent notch integrated bandpass filter design.

A clear passband is observed from 1.0 to 4.6 GHz, with the insertion loss remaining better than −3 dB throughout the passband. The filter also demonstrates satisfactory out-of-band rejection, providing a suitable baseline for the subsequent notch-integrated bandpass filter design.

### 2.3. Design of a Notch Bandpass Filter Based on SSPPs

Defected ground structures (DGSs) are formed by etching periodic or aperiodic patterns into the ground metallization of a planar transmission line. This perturbation distorts the surface current distribution and modifies the local electromagnetic boundary conditions. As a result, the effective inductance and capacitance of the transmission line are altered. For microstrip implementations, the introduction of a DGS typically increases both the equivalent inductance and the equivalent capacitance.

The earliest defected ground structure is the dumbbell-shaped DGS [24]. It consists of two rectangular defect regions that are coupled through a narrow gap and connected by a thin slot etched in the ground plane. DGSs are attractive because they can provide bandstop behavior without requiring a large periodic array. A small number of DGS cells are often sufficient to obtain pronounced rejection while keeping the circuit footprint small. In addition, a single DGS element features a simple geometry that is easy to fabricate, and its equivalent circuit model can be conveniently established and extracted. Moreover, the slow wave effect can be achieved with only one or several cells, which simplifies practical implementation. Motivated by these advantages, this section introduces a DGS into the proposed bandpass filter to generate an in-band notch. The resulting notch enables targeted suppression at a specified interference frequency.

**Figure 6:**
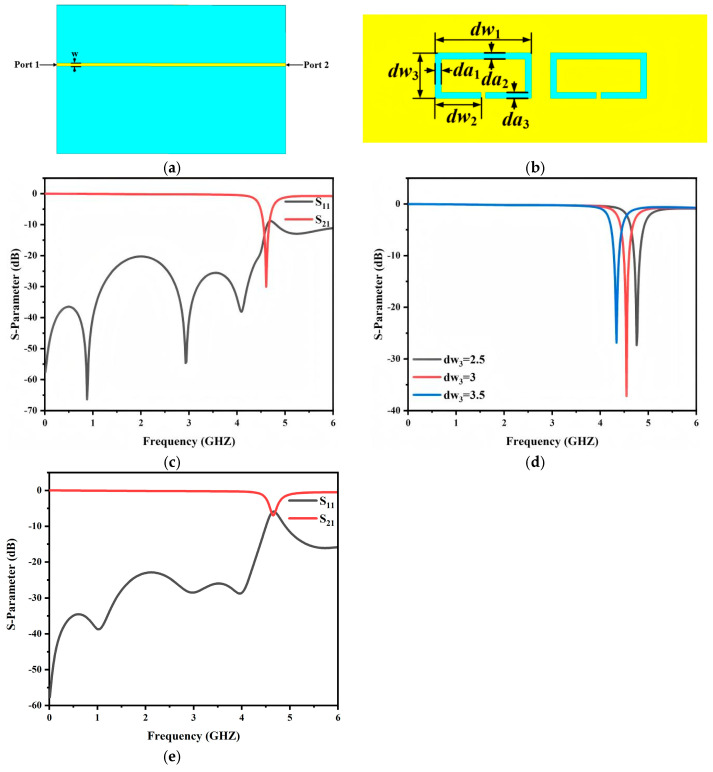
Schematic diagram of the proposed annular opening groove DGS: (**a**) front view, (**b**) back view; (**c**) S−parameters; (**d**) influence of different dw_3_ values on S_21_; (**e**) S−parameters of an open slot.

Despite the aforementioned advantages, the conventional dumbbell-shaped DGS still occupies a relatively large etched area on the ground plane due to its two-slot configuration. This becomes a limiting factor when further miniaturization and high integration are required in the proposed filter. To alleviate this constraint, an open-slot annular DGS is adopted, as shown in Figure 6b. Compared with the traditional dumbbell topology, the proposed structure exhibits a more compact layout and requires less ground plane area while maintaining the desired bandstop characteristic. In addition, the slot length and slot width provide effective tuning parameters for accurately controlling the notch frequency, thereby enabling targeted suppression near the specified interference band within a constrained footprint.

To clearly demonstrate the electromagnetic characteristics of the proposed annular DGS, a 50 Ω microstrip feed line with a width of 0.72 mm is introduced at the input side of the simulation model, and wave ports are assigned at both ends of the microstrip line, as illustrated in Figure 6a. The annular defect is etched on the backside ground metallization, as depicted in Figure 6b. Consequently, the proposed DGS improves ground plane utilization and supports further miniaturization and integration while preserving the required filtering performance.

Figure 6c plots the simulated S_11_ and S_21_ of the proposed DGS. A pronounced notch is clearly observed in the target band. It exhibits deep attenuation, which confirms strong rejection capability. This response effectively suppresses the undesired spectral components at the specified frequency.

To investigate the influence of the slot DGS parameters on the notch behavior the transmission responses under different dw_3_ values are presented. As shown in Figure 6d increasing dw_3_ shifts the notch frequency toward the lower band. This trend can be interpreted using an equivalent circuit model. A larger dw_3_ effectively extends the slot line length and increases the effective slot area. The corresponding series equivalent inductance therefore increases. Since the resonant frequency decreases with increasing inductance, the notch frequency moves to a lower value accordingly [25]. Hence the notch frequency can be controlled within a certain range during the design stage by adjusting dw_3_. Moreover, an appropriate choice of dw_3_ enables accurate notch placement while preserving a compact layout and simple implementation, thereby enhancing the applicability and tuning flexibility of the proposed DGS in microwave circuits.

Furthermore, the number of annular slots directly affects the notch characteristics. Each annular slot introduces a localized resonance that contributes to the suppression within the passband. When only one slot is used, as shown in Figure 6e, the notch suppression depth is limited to approximately −7 dB. Introducing a second slot, as in Figure 6c, provides an additional resonance, enhancing the suppression depth to −25 dB. From a theoretical perspective, the number of slots is also related to the number of SSPP resonant cells in the bandpass filter. Since the designed filter employs two SSPP resonators, using two annular slots ensures mode matching between the resonators and the notches, achieving efficient suppression at the target frequency. Using more than two slots may lead to undesired coupling between resonances, complicating the notch response and potentially degrading the overall passband performance. Therefore, considering both the notch suppression depth and the resonance alignment with the SSPP cells, two annular slots are adopted in this design.

**Figure 7:**
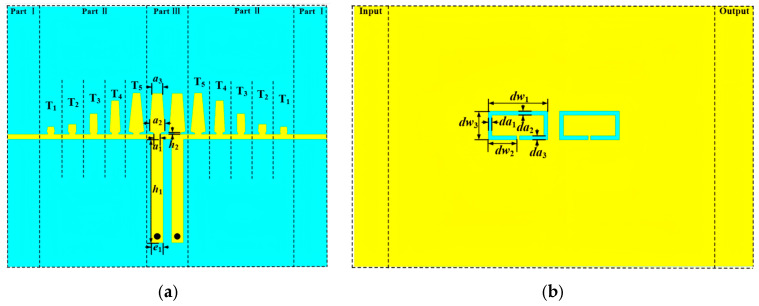
The designed notch bandpass filter circuit structure: (**a**) front view; (**b**) back view.

By etching the proposed DGS onto the ground plane of the bandpass filter described in the previous section, a notch response is incorporated into the original bandpass characteristic. The final notch bandpass filter is illustrated in Figure 7, showing both the top and bottom layouts. The prototype is implemented on a Taconic RF 60 substrate with a relative permittivity of 6.15, a thickness of 0.508 mm, and a copper thickness of 0.018 mm. The bottom layer is fully metallized and serves as the ground plane. Two annular-slot DGS elements are positioned on the backside beneath the SSPP resonator region.

**Figure 8:**
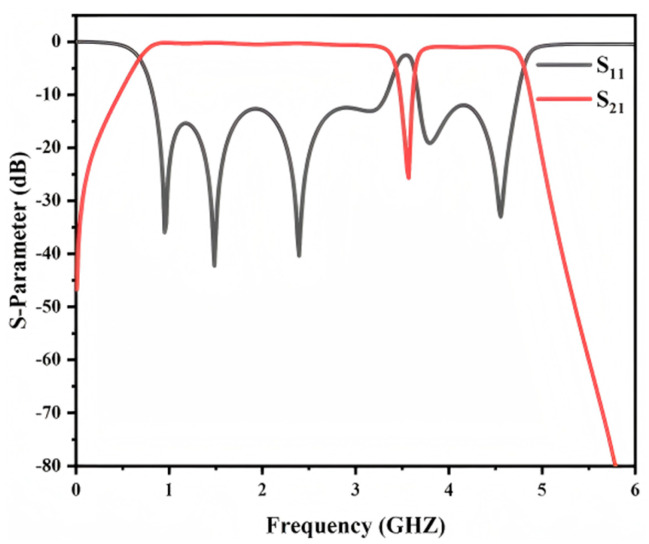
Simulation S−parameters of the notch bandpass filter.

Figure 8 presents the simulated S-parameters of the proposed notch bandpass filter over the full frequency range. Two passbands are obtained from 0.67 to 3.40 GHz and from 3.67 to 4.77 GHz. The insertion loss is 0.48 dB at 2.00 GHz and 1.11 dB at 4.22 GHz. The return loss in both passbands remains better than −12 dB. In addition, the etched DGS introduces a deep notch centered at 3.50 GHz with a rejection level of −25 dB. A deep notch of −25 dB is achieved at 3.50 GHz to mitigate interference around the 3.5 GHz band, which is relevant to several wireless and radar related scenarios. These results confirm high in-band transmission and effective suppression at the specified frequency.

**Figure 9:**
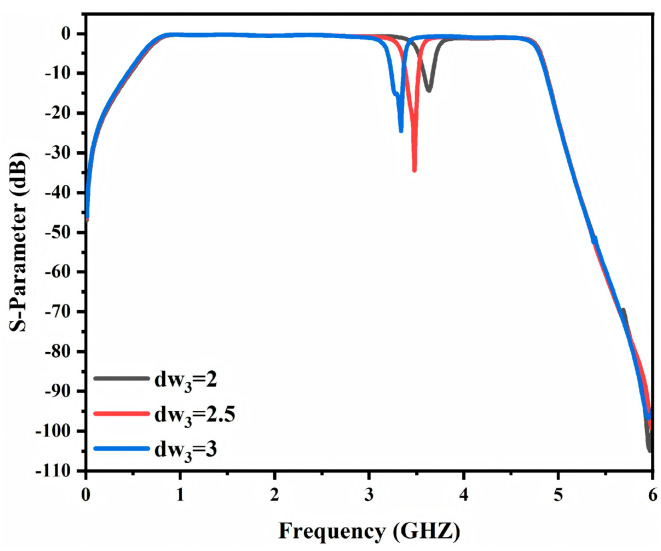
Simulation S−parameters of different dw_3_ notched bandpass filters.

Furthermore, the dispersion-based analysis indicates that varying the effective area of the defected ground structure changes its equivalent inductance. This shifts the resonant frequency of the DGS and consequently tunes the notch frequency. As shown in Figure 9, the notch location changes as the DGS parameter dw_3_ is adjusted. When dw_3_ increases from 2 mm to 3 mm, the notch frequency shifts from 3.6 GHz to 3.3 GHz. Therefore, the notch position can be precisely predefined during the design stage through appropriate selection of the DGS geometrical parameters to satisfy different application requirements. This feature enables a more adaptable bandpass filter design for suppressing designated interference frequencies.

**Figure 10:**
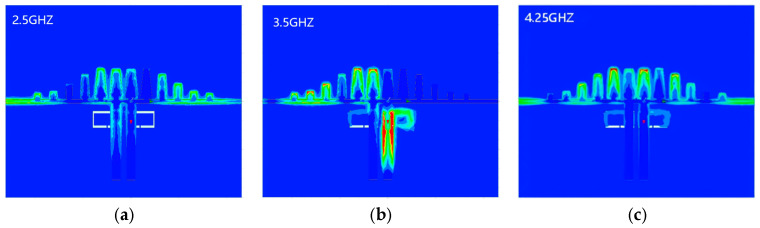
Electric field distribution at different frequencies: (**a**) 2.5 GHz, (**b**) 3.5 GHz, (**c**) 4.25 GHz.

Figure 10 shows the simulated electric field distributions at representative frequencies to clarify the operating mechanism of the proposed notch bandpass filter. At 2.5 GHz and 4.25 GHz within the passband, the field is mainly confined along the transmission path and propagates with weak attenuation. In contrast, at 3.50 GHz, the field is strongly coupled to the defected ground structure and rapidly attenuated. This behavior indicates that the DGS introduces a resonant rejection at the notch frequency and suppresses wave transmission through the filter. Consequently, the 3.50 GHz component is effectively blocked, while the passband signals are maintained.

## 3. Measure and Discussions

**Figure 11:**
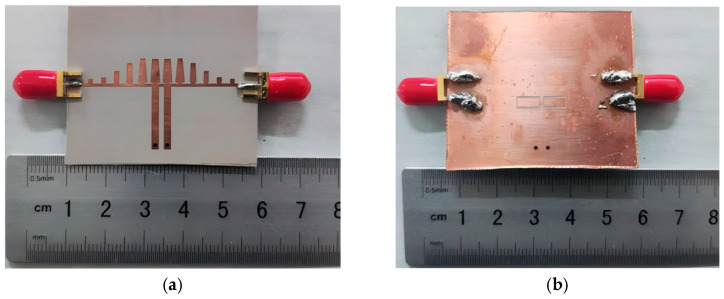
Notch bandpass filter prototype (**a**) front view, (**b**) back view.

**Figure 12:**
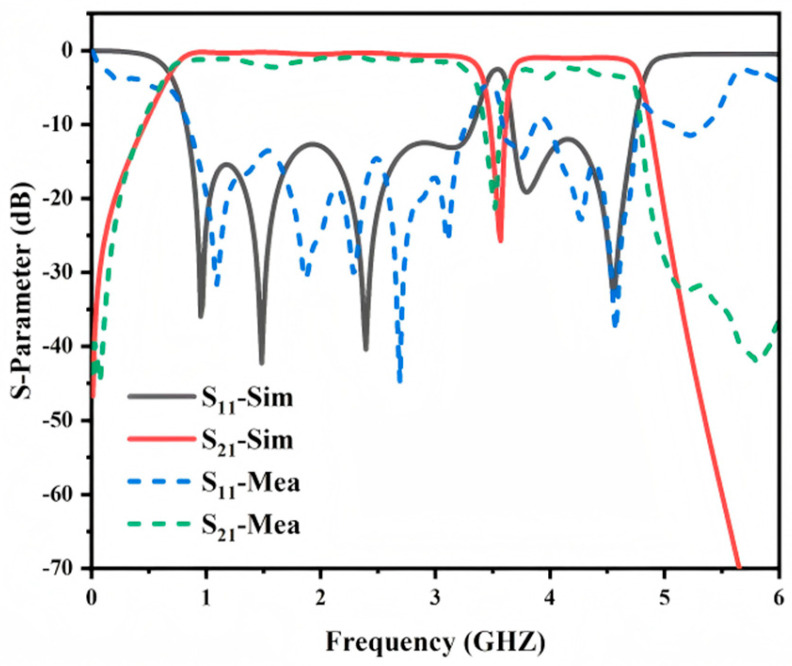
Simulation and measured S−parameters of notch bandpass filter.

To evaluate the practicality of the proposed notch bandpass filter, a prototype was fabricated using standard PCB processing techniques. Photographs of the fabricated sample are provided in Figure 11, and the measured S-parameters are shown in Figure 12. The measured passband locations and overall response trends agree well with the simulations. In the measurements the insertion loss is about 1.5 dB in the first passband and about 2.5 dB in the second passband. The notch frequency is also consistent with the simulated prediction, and a rejection level of approximately −25 dB is obtained at 3.50 GHz. The return loss within the passbands remains better than −10 dB.

The slight differences between the measured and simulated results are mainly attributed to non-ideal practical factors, which become more pronounced at higher frequencies. The deviations are primarily observed in the upper cutoff frequency, passband ripple, and insertion loss.

First, the notch is realized by etching an annular slot in the ground plane. This defected ground structure disrupts ground current continuity and introduces additional radiation and conductor losses, which inherently increase the insertion loss compared with an ideal complete-ground structure. Second, manufacturing tolerances may further influence the performance. The height of the short-circuit stub SSPP resonant unit directly determines the asymptotic frequency of the dispersion curve and therefore affects the upper cutoff frequency. Similarly, the unit height of the transition section impacts impedance matching conditions, leading to variations in passband ripple and insertion loss. In addition, soldering of the SMA connectors and measurement uncertainties may introduce slight impedance mismatch and parasitic effects, contributing marginally to the overall discrepancy between simulation and measurement.

Nevertheless, these non-ideal factors only cause moderate shifts and do not alter the fundamental operating principle of the filter. The measured response remains in good agreement with the simulated results. Overall, the proposed SSPP-based notch bandpass filter successfully reproduces the expected passband and notch characteristics, demonstrating practical feasibility for microwave applications.

Table 1 compares the proposed design with previously reported notch filters. The results indicate that the proposed notch bandpass filter offers a compact footprint, low in-band insertion loss, strong notch rejection, and an independently controllable notch frequency during the design stage.

## 4. Conclusions

In this paper, a notch bandpass filter based on artificial surface plasmon polaritons is presented and systematically analyzed. The bandpass response is realized by an SSPP transition section and SSPP resonant units, which enable smooth mode conversion from the microstrip line to the SSPP transmission line and maintain stable in-band propagation. An annular slot defected ground structure is developed from the conventional dumbbell-shaped DGS to introduce an in-band notch. The notch frequency can be independently set during the design stage by tuning the DGS geometrical parameters, with minimal impact on the passband edges. A prototype is fabricated and measured to validate the design. Measured results agree well with simulations. Two passbands are obtained from 0.67 to 3.40 GHz and from 3.67 to 4.77 GHz. The insertion loss is 0.48 dB at 2.00 GHz and 1.11 dB at 4.22 GHz. The return loss in both passbands adjacent to the notch is better than −12 dB. A deep notch centered at 3.50 GHz provides −25 dB rejection. The proposed filter shows application potential in high performance microwave and millimeter-wave communication systems. It is particularly suitable for suppressing specific interference frequencies in 5G platforms.

## Figures and Tables

**Table 1:** Performance comparisons with previous work.

References	Suppression Depth	Insertion Loss	Notch Frequency Adjustable	Size (λg × λg)	Structure Type
[15]	−17 dB	1, 1.5	NO	0.84 × 0.42	SSPPs
[16]	−14 dB	2.5, 3	NO	3.15 × 0.38	SSPPs+CPW
[17]	−25 dB	2.5, 2.5	NO	1.51 × 0.42	SSPPs+DSPSL
[19]	−18 dB	3, 2	NO	5.26 × 0.81	SSPPs+CPW
[20]	−28 dB	1.12, 1	NO	2.07 × 0.63	SSPPs+CPW
This work	−25 dB	1.5, 2.5	YES	1.05 × 0.84	SSPPs+DGS

λg: Waveguide wavelength.

## Data Availability

The original contributions presented in this study are included in the article. Further inquiries can be directed to the corresponding author.
